# Total knee arthroplasty in valgus knee deformity: is it still a challenge in 2021?

**DOI:** 10.1007/s12306-021-00695-x

**Published:** 2021-02-15

**Authors:** D. Alesi, A. Meena, S. Fratini, V. G. Rinaldi, E. Cammisa, G. Lullini, V. Vaccari, S. Zaffagnini, G. M. Marcheggiani Muccioli

**Affiliations:** 1 2nd Orthopedic and Traumatologic Clinic, IRCCS Istituto Ortopedico Rizzoli, Via G.B. Pupilli 1, 40136 Bologna, Italy; 2 University of Bologna, Bologna, Italy; 3 VMMC and Safdarjung Hospital, Central Institute of Orthopedics, New Delhi, 110029 India; 4 UO Medicina Riabilitativa e Neuroriabilitazione, IRCCS Istituto delle Scienze Neurologiche, Via Altura 3, 40139 Bologna, Italy

**Keywords:** Valgus knee, Total knee arthroplasty, Surgical approach, Soft tissue balance

## Abstract

Total knee arthroplasty in valgus knee deformities continues to be a challenge for a surgeon. Approximately 10% of patients who undergo total knee arthroplasty have a valgus deformity. While performing total knee arthroplasty in a severe valgus knee, one should aware with the technical aspects of surgical exposure, bone cuts of the distal femur and proximal tibia, medial and lateral ligament balancing, flexion and extension gap balancing, creating an appropriate tibiofemoral joint line, balancing the patellofemoral joint, preserving peroneal nerve function, and selection of the implant regarding constraint. Restoration of neutral mechanical axis and correct ligament balance are important factors for stability and longevity of the prosthesis and for good functional outcome. Thus, our review aims to provide step by step comprehensive knowledge about different surgical techniques for the correction of severe valgus deformity in total knee arthroplasty.

## Introduction

Total knee arthroplasty (TKA) in valgus knee deformities continues to be a challenge in prosthetic surgery. Approximately 10% of patients undergoing total knee arthroplasty have a valgus deformity, i.e., valgus of > 10° [1]. Osteoarthritis is the most common cause of valgus deformity, even though other events and diseases such as post-traumatic arthritis, rheumatoid arthritis, rickets, and renal osteodystrophy can lead to a valgus knee [2]. The valgus arthritic knee is often a complex deformity characterized by hypoplastic lateral condyle, lateral tibial plateau bone loss, external rotation deformity of the tibia, femoral and tibial metaphyseal valgus remodeling, and patellar malalignment [3, 4]. Moreover, tightening of lateral soft tissue structures including ilio-tibial band (ITB), lateral collateral ligament (LCL), posterolateral capsule (PLC), posterior cruciate ligament (PCL), and popliteus tendon (POP) and concomitant instability can be found in valgus knee deformity. In rare cases, the lateral head of the gastrocnemius and the long head of the biceps femoris are also affected. These soft tissue contractures are responsible for lateral patellar subluxation, patellofemoral maltracking symptoms and represent the prevalent cause of postoperative knee instability [5]. Tightening of lateral soft tissue structures may be associated with attenuation of Medial Collateral Ligament (MCL), and sometimes MCL is incompetent when the deformity is severe. Thus, attaining a well-balanced TKA can be extremely difficult and challenging.

Many classifications are given for valgus deformity of the knee and these are typically based on the severity of the deformity and the extent of soft tissue involvement. Ranawat et al. [6], Krackow et al. [7], Lombardi et al. [3] described a similar classification of valgus malalignment. Ranawat described three grades for valgus deformity (Grade I, Grade II, and Grade III valgus knee). In Grade I, valgus deviation is less than 10°, it is correctable by varus stress test and the MCL is functional and intact. This type accounts for 80% of all valgus knees. In Grade II (15% of valgus knees), the axis deviation ranges between 10° and 20° and MCL is functionally elongated. Grade III, accounting for the remaining 5% of valgus knees, includes an axis deviation higher than 20°, with severe impairment of medial stabilizing elements. In these cases, a constrained implant should be required.

While performing total knee arthroplasty in a severe valgus knee, the technical aspects of surgical procedure, i.e., bone cuts of the distal femur and proximal tibia, medial and lateral ligament balancing, flexion and extension gap balancing, creating an appropriate tibiofemoral joint line, balancing the patellofemoral joint, preserving peroneal nerve function, and selection of the implant regarding constraint should be taken in particular account. Restoration of neutral mechanical axis and correct ligament balance are important factors for stability and longevity of the prosthesis and for good functional outcome.

Thus, our review aims at providing a step by step comprehensive analysis of different surgical techniques for the correction of severe valgus deformity in total knee arthroplasty.

## Surgical considerations

### Preoperative evaluation

Preoperative planning is strongly recommended before all total knee arthroplasties and surgeon must inform treated patients about potential peroneal palsy in cases of severe valgus deformity. Surgical management depends on the extent of deformity.

Preoperatively, every candidate should be clinically evaluated for weight-bearing alignment, flexion contracture, and ligamentous instability along with preoperative radiological assessment including weight-bearing anteroposterior, lateral, and sunrise radiographs as well as measurement of the limb axis deviation with long-standing views of the knee for overall coronal alignment. Radiographs should be evaluated for osseous deformity, patellar thickness and position, alignment of the ipsilateral hip, soft tissue laxity such as medial–lateral opening and/or tibial subluxation, appropriate angles of femoral and tibial resection and the need for augmentation of osseous deficiencies. On the lateral radiograph, any posterior osteophytes should be identified and then removed during surgery as they may hinder the range of motion as well as the soft tissue balance.

### Surgical approach

#### Medial parapatellar approach

In TKA for both varus and valgus knees, medial parapatellar approach is the standard approach and most surgeons prefer this technique. Satisfactory long-term results with this approach have been reported by many authors[1, 3, 4, 7]. The employ of this approach can result in a difficult reach of the posterolateral corner and release of tight soft tissue structures in moderate and severe valgus knee arthritis, in a difficult de-vascularization of the patella if a concomitant lateral release is performed, and there may be a higher potential risk for over-release of medial soft tissues resulting in instability. However, good clinical results have been documented with this approach therefore is considered a good choice for most of the surgeons.

#### Lateral parapatellar approach

The use of a medial parapatellar approach in moderate and severe valgus arthritic knee may present more difficulties in the reach of the posterolateral corner for the releases. However, Buechel [8], Fiddian [9], and Keblish [10] suggested the lateral parapatellar approach (LPA) reporting a better clinical outcome compared to the medial one when performing a TKA for a valgus knee. LPA has been advocated as an alternative to the medial one as it facilitates direct access for the release of tight lateral ligamentous structures with preservation of the medial structures, optimizes patellar tracking, and preserves medial blood supply to the patella, reducing the use of constrained implants [11].

LPA may be associated with technical difficulties and complications, including the possible need of a tibial tubercle osteotomy to achieve an adequate exposure and difficulties in the soft tissue closure after alignment correction. To overcome these complications, other authors introduced a modified LPA [11–14] reporting satisfactory clinical results. However, the lateral approach is still considered a second choice due to its technical difficulties.

### Mechanical alignment and bone resection

In mechanically aligned TKAs, there should be a neutral coronal plane alignment and bone cut should be orthogonal to the mechanical axis. In a valgus knee, both femoral and tibial pathology should be addressed with respective bone cuts to achieve this goal. The normal knee typically has 6° of valgus angle; however, in some cases, after surgical correction and despite achieving this desired 6° of femoro-tibial valgus angle, there may be a residual valgus malalignment [15]. Hence, distal femoral resection should be performed with 2° of over-correction to maximize restoration of the mechanical axis. Therefore 3° of valgus is used to prevent under-correction of the underlying deformity as opposed to the typical 5° to 7° of valgus used for a varus knee [1, 3].

Some authors have reported that under-corrected knees do not behave differently from well-aligned knees, while over-corrected knees with varus deformity showed a statistically higher rate of complications and lower Knee Society Score (KSS). In case of a fixed severe valgus knee, an over-correction of the Hip–Knee–Ankle angle (HKA) should be avoided, especially regarding the tibial mechanical angle. However, a residual valgus angle of more than 6° can induce patellar maltracking [16, 17].

For distal femoral resection, the medial femoral condyle represents a reference point. A minimal amount of bone should be removed from the lateral femoral condyle because of wear and atrophy of this condyle. This will allow restoration of the joint line [3].

In the valgus malaligned knee, the posterior lateral femoral condyle is often deficient and so relying on the posterior condylar axis can result in malrotation of the femoral component. Instead, the anteroposterior (AP) axis (Whiteside’s line), the trans-epicondylar axis, and the tibial shaft axis should be used as reference to achieve correct femoral component rotation [18].

The tibial cut should be orthogonal to the tibial mechanical axis. Before the tibial cuts are made, alignment should be confirmed with the alignment guide because if the planned tibial cut is based on proximal tibial anatomy, this may result in under-correction of the deformity if there is unrecognized extra-articular tibial valgus [19].

### Soft tissue balancing

Soft tissue balancing is not only the key component in achieving good functional results and knee stability following knee arthroplasty but also the most challenging aspect of primary total knee arthroplasty in a valgus knee. Although over the three decades several approaches and soft tissue procedures have been advocated, there is no consensus regarding the structures that need to be addressed during TKR and the order of their release. However, an adequate lateral soft tissue release should be performed to prevent residual valgus deformity and patellofemoral alignment problems [20] avoiding extensive releases that may lead to a high incidence of complications, i.e., residual instability. Partial releases were then introduced as a safe procedure [21] to guarantee a correct soft tissue balancing during TKR. All described technique and clinical outcomes are resumed in Table 1.

**Table 1:** Soft tissue release techniques described for valgus knee correction in TKA and their clinical outcomes

Author	No. of patients	Years of follow-up	Soft tissue release technique	Clinical outcomes
Insall (1979) [21]	220	3–5 years	ITB, lateral capsule, LCL, POP, and posterior capsule along with lateral retinaculum	Postoperative HSS score: (62%) excellent; (28%) good; (4.5%) fair; (5.5%) poor
Whiteside (1999) [24]	231	6 years	LCL, POP, ITB, Posterior capsule	None
Clarke (2004) [26]	24	24–69 months	Pie crust technique of the posterolateral capsule and ITB	mKSS: 97 (range 87–100) mROM: 121° (range 100°–145°)
Ranawat (2005) [6]	35	5 years	“Inside-out” technique PCL, Pie crust technique of the posterolateral capsule and ITB	Modified mKSSc score: 93 points postoperatively mKSSf: 81 points mROM: 110° both preoperatively and postoperatively The mean coronal alignment was corrected from 15° of valgus preoperatively to 5°of valgus postoperatively
Aglietti (2007) [28]	48	5 years	Posterolateral capsule, LCL, ITB	KSSc: 90 points KSSf: 83 points
McAuley (2008) [30]	100 knees	Mean 8.2 years; range 0.1–18.2 years	“Outside-in” releases (LCL and popliteus from the lateral epicondyle followed by deeper structures) in 46 knees “Inside-out” like Ranawat technique in 32 knees	KSS (89 ± 13) when LCL and popliteus tendon preserved KSS (76 ± 24) when both released LCL and POP release increased the revision by 19.9 times LCL and POP release had lower KSS mediolateral laxity (more laxity) than those that had release of neither or one of the two structures
Politi and Scott (2004) [31]	35 knees	Averaged 41.6 months (range 24–67 months)	Cruciform release of lateral retinaculum, PCL partially release only if excessive tight in flexion, release of the ITB and posterolateral capsule	Preoperative valgus averaged 17°, and ROM averaged 10°–107° Postoperative valgus averaged 4.8°, average postoperative ROM was 2° to 110° Stable flexion and extension gaps were achieved in all cases
Mayer (2012) [32]	47 patients (51 knees)	Mean follow-up time was 42 ± 23 months (range 19–107 months)	Excision of the menisci and cruciate ligaments, Posterolateral capsular release as the sole method of lateral release	Preoperative Valgus angle 17.5 ± 4.6 (range 11–31 degrees) Postoperative 6.3 ± 2.2 (range 2–11 degrees) (*p* < 0.0001) Knee score improved from 5.4 ± 17.7 (range 20–61 points) preoperatively to 88.2 ± 15.8 (range 45–100 points) (*p* < 0.0001) Functional score improved from 24.8 ± 24.7 (range 20–80 points) preoperatively to 70 ± 30 (range 20–100 points) (*p* < 0.0001)
Peters (2013) [33]	238 valgus knees		Selective release of the popliteus and PCL. Selective release of the ITB. Rarely release of the lateral posterior capsule and release of the LCL	Valgus knees required more releases than neutral or varus knees (*p* < 0.001) Greater preoperative deformity required a greater number of releases KSS values in valgus knees with zero releases were greater compared with valgus knees with one or two releases

*ITB* Ilio-tibial band, *LCL* Lateral collateral ligament, *PCL* Posterior cruciate ligament, *POP* Popliteus tendon, *HSS* Hospital for special surgery score, *KSS* Knee Society Score, *KSSc* Knee Society Score clinical, *KSSf* Knee Society Score functional, *mKSS* mean Knee Society Score

A very high rate of late-onset instability is reported when the ITB is divided transversely above the joint line, and the LCL and the POP are released from the lateral femoral condyle. Peroneal nerve palsy and/or late-onset instability are reported with large lateral release [22]. Peroneal palsy is the most serious possible complication. Keeping the knee in slight flexion in immediate postoperative period may help to prevent this complication.

In 1979, Insall et al. [23] described a soft tissue balancing technique in which the ITB, lateral capsule, LCL, POP tendon, and posterior capsule were released along with lateral retinaculum; this technique led to a very high rate of late-onset instability.

Whiteside [24] recommended sequential releases of the ITB, POP, LCL, and lateral head of the gastrocnemius even though such release may not be adequate because of late-onset instability. He noticed that the POP tendon needed to be released only in cases of extreme tightness. Moreover, he performed a tibial tubercle transfer when the Q angle (the angle subtended by the quadriceps and patellar tendons) was > 20° although this procedure can lead to non-union and subsequent extensor mechanism problems.

In 2004, Clarke et al. described the “pie crust” technique, in which they release PLC and ITB using multiple horizontal stab cuts, which leads to possible indirect stretching of the LCL. They reported a good range of motion and good functional results according to the Knee Society Scores in 19 knees with correction of the deformity to a mean of 5° (range 2 to 7) at 24 and 69 months follow-up [25, 26].

In 2005, Ranawat et al. [6] developed a less extensive “inside-out” technique of soft tissues release. In this technique, the soft tissues are balanced by using electrocautery in extension to achieve a rectangular gap after resection of femur and tibia. First, they removed marginal osteophytes and PCL was released. Then, the posterior capsule and posterolateral capsular complex was released along the proximal tibial border. POP was not usually released. If the ITB was tight, they performed a “pie crusting” technique by creating multiple small cuts in the ITB to increase the length preserving continuity. Once extension gap was balanced, they achieved equal flexion gap by resection of posterior femoral condyle parallel to the tibial cut. In 35 patients, no cases of delayed instability were reported while implant survivorship was 100% at ten years and 83% at 15 years after implantation of a posterior stabilized or constrained TKA. This technique was used by others with good functional results and correction of the deformity [27].

Aglietti et al. [28] performed “inside-out” release sequence with additional release of LCL by using Pie crusting technique for knee arthroplasty in valgus deformity. They reported good range of motion and good functional outcome in 53 knees at 5 and 12 years follow-up and concluded that selective multiple puncture release of the lateral soft tissues of the knee joint can correct moderate to severe valgus deformities independently from the implant. Peroneal nerve may be at risk when puncturing the posterolateral corner of the knee at the level of the joint line. Clarke et al. [29], in an MRI study, reported that the distance between peroneal nerve and tibial corner was 0.9 to 2.2 cm and that the nerve was always separated from the bone by the interposition of the lateral gastrocnemius so pie crusting should then be safe enough to avoid nerve injuries.

McAuley et al. [30] retrospectively reviewed the results of 100 knees with a valgus deformity of 8° or more treated with different implants and lateral release techniques and reported that resection of the LCL and POP during surgery was associated with high rates of reoperation, possibly due to instability.

Politi and Scott [31] described a technique for posterolateral capsular release in a cruciform pattern using a vertical cut followed by a horizontal cut. The PCL of all the knees was preserved. All knees had a valgus deformity of 15°or more and they reported good stability and deformity correction. They emphasized the need for minimal bone cuts even when almost no bone was resected from the lateral femoral condyle.

Mayer et al. [32] reported results of TKA in valgus deformity using a posterolateral capsular release as the sole method of lateral release. They retrospectively reviewed 42 patients and, in all cases, knees were successfully balanced without the need for further release of other structures.

Peters et al. [33] found that an increase in valgus deformity required more soft tissue releases. Xie K et al.[5] reported that increased severity of preoperative deformity correlated with the need for more soft tissue release but, it did not correlate with the need for lateral retinacular release. Selective soft tissue release for primary valgus TKA was effective without increasing prosthetic constraint.

Medial reconstruction is also an effective approach to deal with the severe valgus knee deformity. Healy et al. [34] and Krackow et al. [7, 35] recommended medial soft tissue advancement combined with lateral soft tissue releases. Currently, the techniques for medial reconstruction consist of MCL advancement by ligament suture and screw-washer combination and MCL advancement with bone plug recession by ligament suture, with a complete prior release of the lateral soft tissue. However, different levels of medial laxity were reported in the post-op due to the ligament suture that was woven into the MCL in the direction of the tendon fibers and to the fact that patients were often affected by osteoporosis, which resulted in failed ligament loading, poor fixation and contributed to the medial instability and recurrent valgus deformities [36]. Considering these facts in 2018, Mou et al. recommended medial femoral epicondyle up-sliding osteotomy to up-slide the osteotomized epicondyle with the MCL origin to provide medial tightening. After medial and lateral balance was achieved, the bone fragment was fixed using hollow screws augmented by washers for bony union. They found this technique as a safe and effective method for the correction of valgus knees, particularly for severe valgus knees.

In cases of severe or rigid valgus deformity, lateral soft tissue release may not adequately balance the knee; therefore, the division of LCL and POP may be required even though this may lead to postoperative instability. Conventional lateral epicondylar osteotomy for the release of LCL and POP tendon can be not enough controlled and may result imprecise [37]. Due to these limitations, sliding osteotomy of the lateral femoral condyle can be performed without computer navigation [38] or with computer navigation [37, 39]. This is a useful technique for soft tissue balancing in TKA with severe valgus deformity where transfer of the femoral condyle with its ligamentous insertions, provides an indirect release of the LCL, POP tendon, and part of the PLC.

Brilhault et al. [38] described a sliding osteotomy of the lateral epicondyle for rigid valgus deformities but this approach can improve the risk of subsequent pseudoarthrosis. Mullaji et al. [37] used navigation for lateral femoral epicondylar osteotomy during valgus TKAs, allowing precise, controlled and quantitative lengthening of the tight lateral soft tissue structures and a proper restoration of soft tissue balance. They used internal fixation with compression screws coupled with large contact surfaces of cancellous bone at the osteotomy site which allowed early postoperative rehabilitation and good union at the osteotomy site. Hadjicostas et al. [39] reported that condylar transfer allows the use of smaller polyethylene inserts since the need of PCL release is minimum and bone stock is preserved for eventual future revision surgery. Moreover, rotation of the femoral component, determined by balancing of the femoral flexion gap, improves patellofemoral tracking and produces collateral ligaments balance. Li et al. [40] reported that lateral femoral sliding osteotomy is an effective and safe technique for TKA with severe valgus deformity over 20° and satisfactory outcomes were achieved without the use of constrained implants and with a low risk of common peroneal nerve injury. However, drawbacks such as non-union, malunion of the osteotomized fragments, increased surgery time, and delayed mobilization are associated with medial and lateral condyle osteotomy.

Computer-assisted surgery can provide the correct positioning of the lateral epicondyle in order to balance the knee on the lateral side without ligament release. It also provides information for the correct balancing of flexion, extension, and rotational alignment and records of mediolateral laxity in flexion and extension can be obtained with the navigation system. However, repeated measurements during surgery add more time to the procedure [39].

There is not enough evidence in the literature to conclude which is the best technique for lateral soft tissues release. There are neither prospective nor controlled studies on this issue; however, there has been a trend toward minimal release over the past few years, with extensive release being associated with higher instability, loosening, and reoperations. Soft tissues should be carefully released, with the goal of retaining partial integrity of the lateral soft tissue stabilizers.

### Implant choice

There is no consensus on the degree of implant constraint that should be used in valgus knee arthroplasty. Both cruciate-retaining (CR) and cruciate-sacrificing TKA implants have been used with satisfactory clinical results. On one hand, some authors [1, 3, 22, 41] suggest PCL-substituting implant designs to avoid concerns with PCL balancing and deal with a potentially abnormal native ligament. On the other hand, other authors [3, 4, 7, 30, 31] believe cruciate-retaining designs are to prefer to preserve condylar bone in the event of further revision surgery especially among younger patients. Moreover, some authors employed primary constrained components both with and without stem extensions [22, 41].

The choice of implant, however, must be based on the degree of joint instability and the presence of bone defects.

For Grade I valgus knees, CR implants can be used, but proper bony resections with adequate soft tissue balancing are necessary for TKA long-term survival.

In presence of laxity on the coronal plane, due to MCL insufficiency, a greater constraint such as posterior-stabilized (PS), condylar constrained knee (CCK) or hinged implants should be used instead of CR implants to achieve appropriate stability and deformity correction [42]. When the coronal deformity is mild (< 10°) but the MCL tension is inadequate, PS implant can be used. However, in the case of more severe deformities, the mechanical stresses absorbed by the polyethylene cam can lead to premature implant wear and early loosening. For this reason, many surgeons rely on a CCK implant, which has a larger cam and can adopt stems that distribute and dissipate joint stresses along the metaphysis and the diaphysis, with the drawback that it is necessary to remove a larger portion of femoral intercondylar bone to accommodate the femoral box, which decreases the remaining bone stock available for revisions.

CCK implants, when used in the arthritic valgus knee, show good results at mid-term follow-up, with a survival rate of around 97% [22, 43, 44], also without the use of stem extension [41], but little is known about their performance beyond 10 years.

In the case of elderly patients with severe ligamentous insufficiency and multiplanar instability, major bone defects, hyperlaxity, valgus deformity greater than 20° or rheumatoid arthritis, a hinged implant should be the choice, preferably coupled with a fixed bearing because, as suggested by Gehrke et al., the latter improves patellar tracking in the valgus knee [45, 46].

Unfortunately, although hinged implants provide optimal stability in severe valgus deformities with multiplanar instability, they are subject to several limitations, including the need to cement long stems into the tibia and femur, which can be an obstacle to removing them during revisions, and the not negligible risk of loosening or rupture of the implant.

Regarding outcomes, hinged implants in patients with valgus deformity showed a survival rate of 79% at 13 years follow-up [46]. Furthermore, the combination of a valgus deformity with an age at the time of surgery of less than 60 years showed a survival rate of only 64% after 13 years of follow-up comparing to patients with pre-existing varus deformity and age over 60 years at the time of surgery, where it was 95% after the same period of time[45].

When possible, therefore, it is advisable to use the lowest possible degree of constraint to achieve optimal stability.

## Conclusion

The surgical treatment of valgus knee deformity can still present a number of unique challenges. Multiple surgical techniques have been described to treat this dysfunction with satisfactory clinical results; moreover, the adherence to a stepwise approach to deformity correction and implant choice is recommended.
